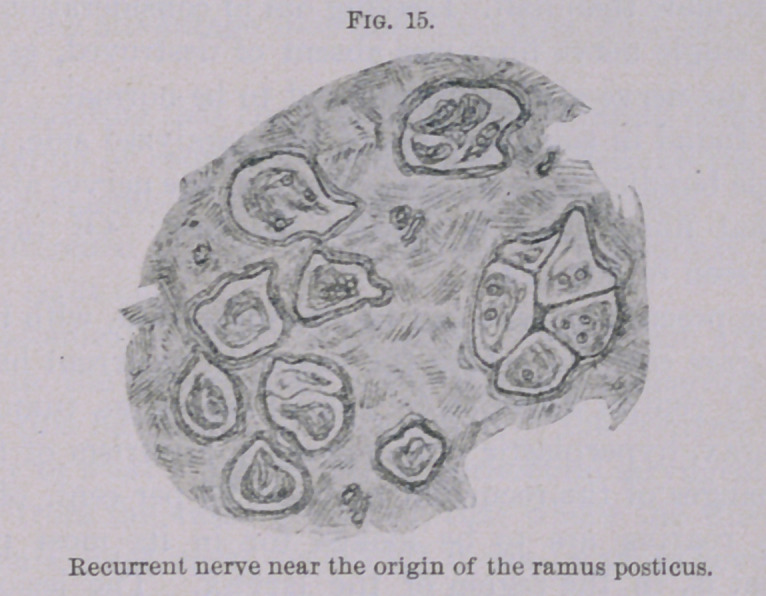# Investigation of the Pathogenesis of Roaring (Hemiplegia Laryngis)

**Published:** 1902-08

**Authors:** M. H. J. P. Thomassen

**Affiliations:** Utrecht, Netherlands


THE JOURNAL
OF
COMPARATIVE MEDICINE AND
VETERINARY ARCHIVES.

VOL. XXIII. AUGUST, 1902. No. 8.

### INVESTIGATION OF THE PATHOGENESIS OF ROARING (HEMIPLEGIA LARYNGIS).

BY PROFESSOR M. H. J. P. THOMASSEN,
UTRECHT, NETHERLANDS.

Translated from Monatshefte für Praktische Thierheilkunde for the JOURNAL.

BY L. VAN ES, M.D., V.S.,
MOBILE, ALABAMA.

WITH reference to the hereditary character of laryngeal roaring, I believed that an investigation of the central nervous system, especially of certain centres of the medulla oblongata, should not be neglected; also the spinal accessory nerve of a few horses was the object of a microscopic examination because it appeared to me very probable that it contributes, as a whole or as a part, to the formation of the recurrent nerve.

I will return to this at another place and at another time.

In the meantime it soon turned out that the changes were limited to the periphery and almost exclusively to the recurrent nerve.

First of all I had the opportunity to conduct an extensive investigation in horses which suffered from spontaneous roaring, and later in those in whom the ailment had been brought about experimentally in various ways. With the exception of the cases in which the whole recurrent nerve was examined microscopically, the material used from other roarers was the peripheral part of the nerves, which were still attached to the right and left of the larynxes forwarded to me.

The results obtained were in the most of the cases tolerably the same, so that it does not seem to me as risking too much in reaching conclusions as to the nature and form of the disease based upon my observation at this time.

Even if the question of etiology is not fully solved, the following facts in that line will bring us a great deal nearer to our purpose.

CASE I.—A brown mare, native breed, about fifteen years old, was purchased for experimental purposes. According to the story of the owner the horse was affected with roaring only a few years.

On the arrival of the horse, and also on the day following, roaring was present in such a degree, even when standing quietly in the stable, that I found it necessary to perform tracheotomy. Judging by the roaring I suspected that its cause in the case under consideration was not to be looked for in the form of a hemiplegia, but of a tumor or a deformity of the larynx.

On a closer examination it soon became clear that the intense sound of stenosis was entirely due to a paralysis of the left half of the larynx.

Laryngotomy performed on the animal revealed the left arytenoid cartilage, immovably in the cadaver position. After the patient had breathed through the tracheotomy tube for a few days, a cork was inserted in the opening, and it soon became evident that the animal not only had ceased to roar when at rest, but that roaring could be detected only very slightly after a preceding movement in trot.

The arytenoid cartilage probably had assumed a more lateral position during the period of rest to which the larynx was subjected after the tracheotomy.

Or was a contraction of the right adductors, in consequence of a vagus lesion on the left side, to be assumed to be the cause of the severe roaring, as has been observed by French authors in man?

As an experiment both the muscular branches of the accessory nerve were cut through, by which the sternomaxillaris was placed out of function on both sides. It has recently been asserted that this has not the least influence on the degree of roaring.

There were no changes to be observed in the frequency of the respiration or the pulse in the horse, which was used a few times for clinical instruction. The horse was killed, and first of all the left recurrent nerve and vagus were critically examined throughout their whole course.

Both nerves were free everywhere; nowhere a trace of adhesions with the surroundings or of any compression by enlarged lymph nodes. They were removed in toto and hardened in

Müller's fluid. The left spinal accessory nerve was treated in a similar manner. The medulla oblongata was first hardened in formalin, and after that in alcohol.

In the excised larynx the following changes were noticed:

The upper edge of the left arytenoid cartilage stood lower and more toward the middle than that of the right side. Most muscles of the left half of the larynx were markedly atrophic. The posticus muscle was degenerated in such a high degree that there only remained a few muscle bundles of the upper third; the lower two-thirds formed nothing but a fibrous plate. The left half of the transversus had entirely wasted. Of the crico-arytenoideus lateralis not a sign was to be found. The least atrophied was the thyro-arytenoideus superior, but the muscle bundles, still intact,

FIG. 1.

Vagus and recurrent nerves in the most anterior portion of the chest. (United in one sheath.)

had an extremely pale color. The crico-thyroideus was not changed, and its volume and its color were similar to the muscle on the right.

The muscles on the right half were red and of a normal volume. The medulla oblongata was examined microscopically first. With reference to the heredity of the disease, the possibility of lesions of the central nervous system had to be reckoned with. After treating the sections according to Nissl's method, the vagus and accessory centres, and more particularly the nucleus ambiguus, received attention in the first place. All ganglia cells by which those centres are formed turned out to be perfectly normal.

Neither did sections of the spinal accessory nerve, stained after Weigert's method, show the least change.

Now came the turn to the vagus and recurrent nerves, which had been hardened in Müller's fluid. The greater part of both nerves was sectioned, and the sections stained after the method of Weigert-Pal. The cuts (Figs. 1 to 5) give a true picture of the preparations. Figs. 1 and 2 were taken with obj. A and ocular I. Figs. 3, 4 and 5 with obj. A and ocular IV. (Zeiss). The illustrations show plainly the intact nerve fibres to be of different size in the normal as well as in the atrophic bundles.

The sections, which were taken from the lower cervical and foremost thoracic region of the vagus at a place where this nerve is still united with the recurrent, showed that the vagus was still quite normal. The recurrent, which in Fig. 1 is situated below, at the right, with its six bundles, which are almost exclusively formed

FIG. 2.

Vagus and recurrent nerves, partly separated.

by thick nerve fibres, can be easily distinguished from the vagus. In preparations taken from the vagus immediately after the giving off of the recurrent in the thoracic cavity, and treated by Pal's method, one observes a couple of bundles, which for a part are considerably degenerated. (Fig. 2.)

In its entire thoracic portion the recurrent does not show the least change, as is evident from Fig. 2 and especially from Fig. 3. The latter section (Fig. 3) shows first of all six bundles to the right, which consist of large nerve fibres, composed of perfectly normal myeline sheaths and normal axis cylinders. The four bundles situated to the left, and almost entirely composed of thin fibres, show themselves in the main to be normal. The section of the recurrent at the upper part of the neck (Fig. 4), however, presents a different picture. Parts of the nerve fibres are quite atrophic, and in their place connective tissue is found. In the vicinity of the larynx, as is shown in Fig. 5, almost none of the fibres have remained. In some bundles there are still two or three left. The bundles composed of fine fibres have suffered the

FIG. 3.

Recurrent nerve in the lower part of the neck.

most. The question now is whether we have to deal with a simple secondary process of degeneration or with the consequences of a neuritis?

My opinion is that the first view should be accepted, because the recurrent shows, some months after its resection, in all its

FIG. 4.

Recurrent nerve in the upper part of the neck, partly degenerated.

peripheral parts, the same changes as are to be observed in the worst cases of spontaneous roaring.

In Fig. 3 one sees to the right six bundles composed exclusively of large fibres, and to the left four bundles, which for the greater part are made up of smaller, probably sensitory, nerve fibres. This difference in the structure, according to my views, points to a different origin of the two halves, an anatomical problem into which I will penetrate no further at this place.

CASE II.—A five-year-old gelding suffered for several months from roaring. The trouble followed a case of benign strangles. The horse roared in such a degree that the owner decided to have it operated on. In January I performed arytenoidectomy, after which the healing process seemed to run a normal course, so that the horse could be discharged in about three weeks. After six weeks no roaring could be detected during light work, so that for the time being a complete cure had been obtained. In the month of April following, difficulty in swallowing suddenly developed in the horse to the extent that feed and water partly regurgitated

FIG. 5.

Recurrent nerve near the larynx, extensively degenerated.

and partly found its way into the trachea, so that after tracheotomy a portion ran out of the tube. As was to be expected, foreign body pneumonia developed. The horse was killed on May 23d, and I had the opportunity to perform a careful autopsy. In the first place the vagus and the recurrent of the left side were examined in their whole course. Nowhere was there to be found anything pointing toward abnormal pressure, and no swelling of lymph nodes existed. Both nerves were free everywhere. The recurrent was removed entirely and the vagus as far as the jugular foramen, and immediately placed in Müller's fluid. Alongside the epiglottis, in the upper folds of the laryngeal mucosa, and also in the nasal cavity, food particles were found; in the lungs necrotic foci, as was to be expected. The removed larynx showed no traces of the extirpation of the arytenoid cartilage. The mucosa was quite even, so that there was almost nothing to be seen of the scar. The opening of the larynx was nearly normal; no retraction of the cicatrix had taken place. There was further to be noticed considerable atrophy on the left side. The most atrophic

FIG. 6.

Recurrent nerve 65 cm. from the larynx.

were the posticus and transversus muscles, so that fibrous tissue had taken the place of muscle fibres. The lateralis muscle also was quite atrophic. The superior and inferior muscles had retained their normal volume almost entirely, but had a conspicuously pale color. They were not examined microscopically.

FIG. 7.

Recurrent nerve 40 cm. from the larynx.

Hardened in the proper manner the sections were stained after the Weigert-Pal method. The following changes were now to be observed:

The recurrent nerve was perfectly normal throughout the whole thoracic cavity, and as far as half of the neck, as can be seen from

Fig. 6 (a cross-section of the nerve at a distance of 60 to 65 cm. from the larynx; here, too, one sees six bundles entirely made up of coarse fibres, and five bundles which are largely composed of finer, probably sensitory, fibres). At a distance of 40 to 45 cm. from the larynx a few fibres are absent, and a beginning degeneration can be plainly observed. A few bundles which probably consisted of fine fibres are entirely degenerated.

In the part situated 5 cm. from the larynx only a few fibres are found, sometimes two or three in one bundle, while just before the place where the twig for the posticus muscle is given off nothing is to be seen of the nerve elements. Fig. 8 shows in the nerve bundles nothing but connective tissue; nerve fibres are entirely absent. Considering the difficulty in deglutition, I hoped to find

Fig. 8.

Recurrent nerve near the larynx.

considerable changes in the vagus. The cervical and thoracic portion proved to be normal on microscopic examination, however.

Case III.—A large, brown, fifteen-year-old mare, bought by me from a drayman, for whom she had to do heavy work. For two years she breathed through a tracheotomy tube, on account of the high degree of roaring, caused by laryngoplegia. The horse was killed by stabbing the heart. The vagus and the recurrent of the left side were followed up minutely, but throughout their whole course not a sign of compression or stretching was found. Especially that part of the recurrent which is situated behind the aorta, and between this artery and the trachea, was as normal in circumference as in front and behind the place mentioned. The left vagus and recurrent, along with the medulla oblongata, were removed and placed in a formalin solution. From the right nerves only a small portion from the middle of the neck was removed. The peripheral nerves were then hardened in Müller's fluid, the medulla in alcohol.

FIG. 9.

Recurrent nerve from the lower part of the neck.

On the left side of the larynx I observed considerable hemi-atrophy. Seen from above, the left arytenoid cartilage was placed lower and more toward the median line than the right one. Of the posticus muscle there remained a few very pale bundles. The

FIG. 10.

Recurrent nerve near the larynx.

left half of the transversus muscle was reduced to a tendinous plate. Of the three muscles remaining the inferior one was the least atrophic. The microscopic examination of both nerves, treated by the Weigert-Pal method, led to the following results:

The vagus nerve was normal throughout. In regard to the recurrent, Fig. 9 shows that throughout the whole thorax it was also normal for the greater part. Here is shown a section of the nerve at the lower cervical portion, in which a couple of bundles show the beginning and one bundle a total degeneration. The latter was probably composed of fine nerve fibres. In the vicinity of the larynx all nerve elements have disappeared. (Fig. 10.) The connective-tissue proliferation (also of the endoneurium) is quite marked, and by which an apparent increase in numbers of the nerve bundles has taken place (by division). In the medulla oblongata, treated by Nissl's method, all ganglia cells of the centres before mentioned were perfectly normal.

Case IV.—A black brood mare of native breed, about nine years old, had for a long time (?) roared in a high degree. She was purchased as an experiment animal for my investigations. During the few days that the animal was kept under observation I discovered that sometimes when moving about quietly in the pasture, or even in the stable, it roared to such an extent that at a distance a loud sound could be heard. When neighing, aphonia became apparent. Other symptoms of disease were not observed in the patient; pulse and respiration were always normal.

The horse was killed by severing the carotid, and the autopsy held immediately after death. As I had convinced myself, during the life of the subject, that as a cause of the roaring a left hemiplegia was to be considered, the left vagus and recurrent were first of all made the object of a minute examination. In this case also not a trace could be found of pressure or constriction of the nerves; they were free throughout and did not adhere to any adjacent organ. Nowhere in the thorax could enlargement of the lymph nodes be observed. The muscles of the larynx were atrophic on the left side, most of all the lateralis and inferior; then the superior, of which a small strip of pale muscle bundles still remained. Although the posticus and transversus did not have their normal volume, they were by no means atrophic to the extent that is usually the case in chronic cases of roaring. The right posticus muscle, however, was also very poorly developed, by which, perhaps, the high degree of roaring can be explained, although the lesion of the left abductor apparently had not yet advanced so far. This is a case in which the most peripheral portion of the recurrent nerve was most involved, by which was caused an idiopathic paralysis of the adductors, giving rise to aphonia before disturbances of respiration could be observed.

By degrees the defect had advanced in a central direction, so that the posticus muscle finally also became involved in the paralysis. The microscopic examination of the nerves gave the following results:

FIG. 11.

Recurrent nerve from the lower part of the neck.

The vagus nerve was perfectly normal. The bundles of the recurrent, composed of coarse fibres, were normal even in the neck. (Fig. 11.) Of the remainder, composed of fine fibres, some consisted exclusively of connective tissue; others were partly degenerated. In the vicinity of the larynx all bundles were considerably

FIG. 12.

Recurrent nerve near the larynx.

changed. (Fig. 12.) Still, there were found more well-preserved nerve fibres at this place than is usually the case in chronic roaring. This corresponds with the unusually little advanced atrophy, which concerns mainly the posticus and the transversus. The especially poor development of the upper half of the right posticus muscle explains the marked stridor, which sometimes could be observed when the animal was at rest or slowly moving about in the pasture.

CASE V.—A black, thirteen-year-old mare, had a few months before become a roarer to such an extent that about six weeks in advance of the purchase it became necessary to perform tracheotomy. As the tracheotomy tube had been removed at the sale I could observe only a slight degree of stridor after the horse had travelled a distance of about one hundred metres in a trot. The mare suffered also from incontinentia urinæ on account of a local affection of the neck of the bladder.

Other signs of paralysis besides the larynx were not observed. The horse was kept under observation for fourteen days, and it

FIG. 13.

Recurrent nerve near the middle of the neck.

soon became evident that a left-sided hemiplegia laryngis was the cause of the dyspnœa. The animal was killed by puncturing the heart, and the autopsy held immedately afterward. In the course of the left vagus and recurrent nothing abnormal was detected by which the origin of a paralysis through mechanical influences could be explained. Both nerves of the left side were hardened as a whole; of the right only a small portion.

The larynx was removed and carefully prepared, and revealed the following changes:

All the muscles of the left half of the larynx, of course, with the exception of the crico-thyroideus, were undoubtedly atrophic, but only in a slight degree. This could be seen most plainly at the superior and inferior muscles, which besides were paler in color. The posticus muscle, on the contrary, had a tolerably normal color, and only its upper half differed in volume from that of the opposite side. All this pointed toward the fact that the process of disease was of a relatively recent date. This is shown by the following communication of Veterinarian Overbeek, of Steenwijk, who had treated the horse: "The animal had shown difficulty in respiration for only a few months. When standing quiet nothing

FIG. 14.

Recurrent nerve near the larynx.

FIG. 15.

Recurrent nerve near the origin of the ramus posticus.

could be detected; as soon, however, as the animal pulled an empty wagon for a distance of about one hundred metres in a trot a loud sound of stenosis during inspiration became evident. The animal, with the exception of the bladder trouble, had not been sick lately, and it certainly had not had pleuropneumonia or strangles during the last few years."

Although the muscles only revealed slight changes, the condition of the recurrent nerve was quite different. However, the nerve mentioned was about normal as far as the middle part of the neck, with the exception of a few bundles (Fig. 13), which were principally composed of fine fibres. In the region of the larynx an almost total degeneration could be observed, as is plainly shown in Figs. 14 and 15. Particularly in the last-mentioned illustration, showing a section of the recurrent nerve at the place where the branch for the posticus muscle is given off, only a few preserved nerve fibres can be found.

In addition the terminal pieces of both recurrents of several larynxes have been subjected to a thorough microscopic examination. The results hereby obtained are, in short, that at the side (left), where a muscular atrophy of some importance is present, the nerve also is almost entirely degenerated, and especially beginning at the place opposite the cricoid cartilage—that is, just in front of the spot where the branchlet for the posticus muscle is given off. A few centimetres in a central direction a few (three or four) fibres can yet be found in each bundle, while higher up they have entirely disappeared.

On the normal side (right) the terminal piece of the recurrent was frequently thicker than on the left. The difference sometimes amounted to more than half. Leaving out of consideration that now and then a single nerve fibre was absent or destroyed, as occurs in all nerves, the nerve always turned out to be normal. Whenever there were found in some muscles of the paralyzed side, in chronic cases, muscle bundles more or less preserved, the nerves also showed more normal fibres than is usually the case. Of this a good example is seen in Fig. 12.

From the preceeding facts can be concluded that, with the exception of the rare cases in which the vagus or recurrent has become diseased in a central direction by special causes, as tumors of the neck or thorax, hyperplastic lymph ganglia, aneurism of the aorta, etc., the changes of the recurrent nerve in 99 per cent. of all cases of nervous roaring are to be looked for in its most peripheral portion, that is, in the region of the larynx. The lesions themselves must probably not be looked upon as a neuritis, but as a simple secondary process of degeneration. The not very important proliferation of the interstitial connective tissue I regard also as secondary. Already at a distance of 30 to 40 cm. from the larynx traces of degeneration can be found. These are then confined, however, to isolated nerve fibres. Sometimes there are found further on, in a central direction, a few bundles entirely or partly degenerated ; this is particularly the case in those which are composed of fine fibres. The thoracic portion of the recurrent nerve was always found to be normal, even in those cases in which the left laryngeal muscles were entirely atrophied and when the disease was of years' standing; also when the disease occurred as a sequel to strangles or pleuropneumonia the thoracic portion of the recurrent proved to be intact. Especially at the place where the recurrent doubles around the aorta, and immediately behind this place, no macroscopic or microscopic changes could be discovered in the the nerve. In the cases in which only an atrophy of the adductors was present, which manifested itself during life by aphonia (frequent in stallions), the nerve degeneration was limited to the extreme terminal portion of the recurrent, especially beginning where the recurrent turns and continues its course under the thyroid cartilage, at the point where the small branches for the posticus and the transversus have already been given out.

Sooner or later the disease progresses in a central direction; the paralysis of the posticus becomes then more prominent, as is manifested by disturbances in respiration. If nerve fibres still remain in the peripheral portion of the recurrent, one also finds greater or lesser portions of the laryngeal muscles intact. This applies first to this and then to that muscle. The supposition that the posticus always has the advantage over the adductors does not hold good in the horse.

Notwithstanding that the process of degeneration is accompanied by proliferation of connective tissue, the recurrent on the paralyzed side has considerably diminished in circumference. Sometimes the circumference of the left recurrent only amounts to half of that of the right one.

The anatomical changes in laryngeal roaring are always more advanced in the nerves than in the muscles concerned. I have discovered this fact before after resection of the recurrent nerve.

Contrary to what I observed in experimental laryngeal paralysis, it is clear, from the cases described, that where the disease affects older animals (over eight years old) it can be of as severe a character as in roaring at a younger age.

There is considerable difference in structure in the twelve or thirteen bundles which form the recurrent. The greater half consists of coarser fibres, the remainder of finer ones, probably partly sensory in nature. It occurs to me that those halves, so easily distinguished from one another, are of a different origin. As this question has merely an anatomical importance, I will return to this at another place.

In this communication I believe I have thrown some light on the nature and character of laryngeal roaring, so frequent in the horse. The etiological question is now in order.

About the exceptional cases in which compression of vagus or recurrent plays a part (tumors, right or left), nothing new is to be said. In man also this form has been closely studied. In the veterinary field we are not as well informed on the toxic forms of laryngeal roaring, among which lead-poisoning occupies a first place. About this I will communicate something later. Entirely in the dark is the causa proxima of the ordinary idiopathic form of hemiplegia laryngis of the horse, in which, as I have shown, the peripheral portion of the recurrent nerve is almost exclusively involved. On this subject I could at this date make some communication; I prefer, however, to await the results of further experiments, in the hope that my present conception of the cause of laryngeal roaring will be confirmed.

## Figures and Tables

**Fig. 1. f1:**
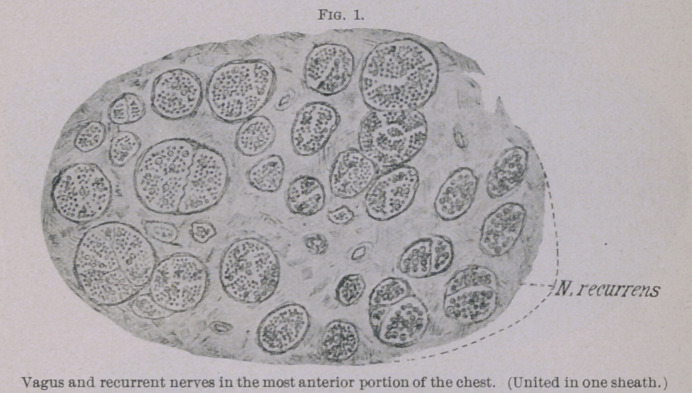


**Fig. 2. f2:**
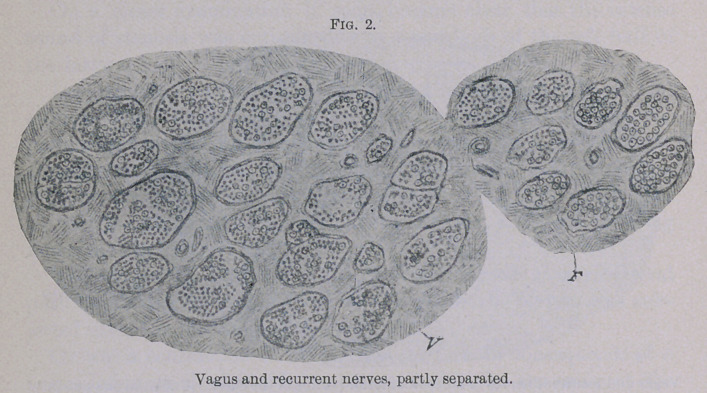


**Fig. 3. f3:**
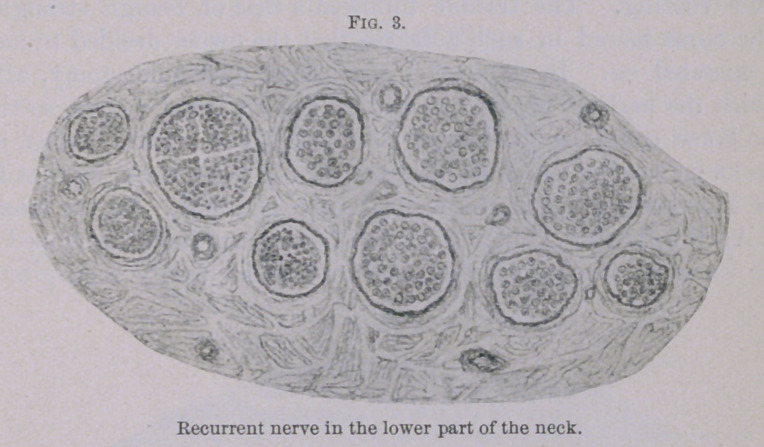


**Fig. 4. f4:**
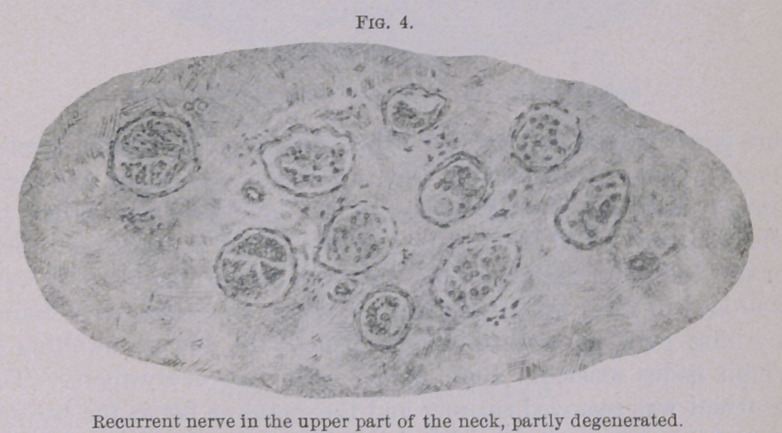


**Fig. 5. f5:**
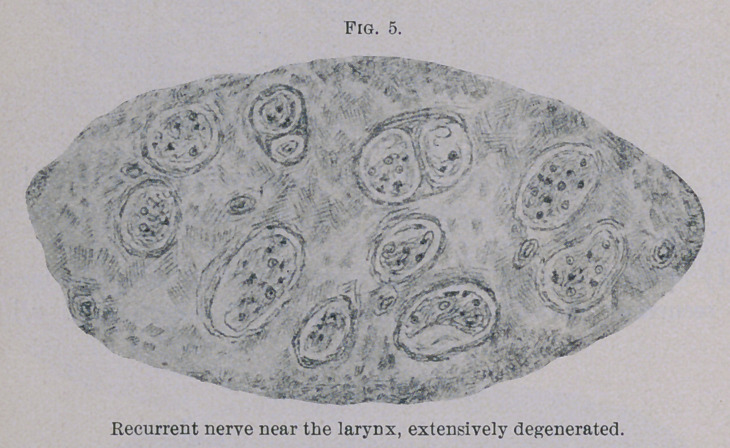


**Fig. 6. f6:**
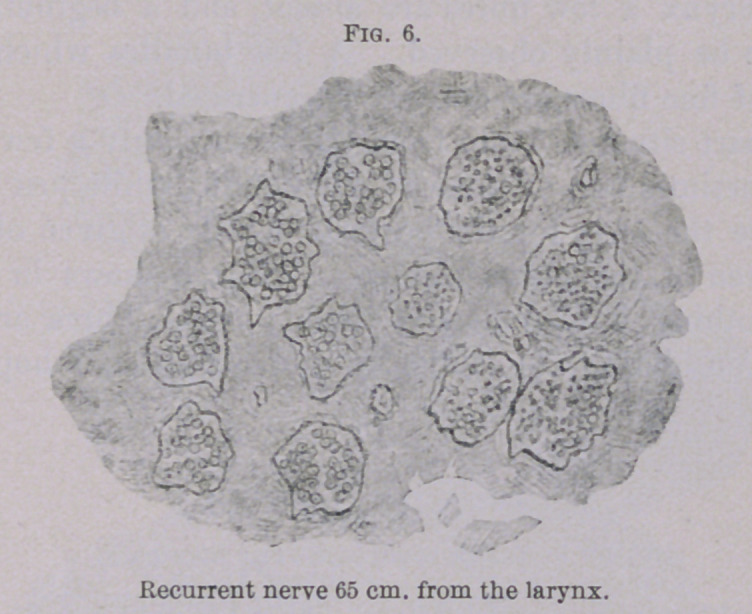


**Fig. 7. f7:**
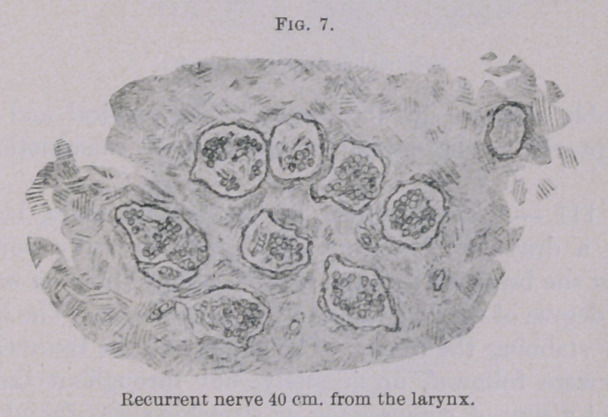


**Fig. 8. f8:**
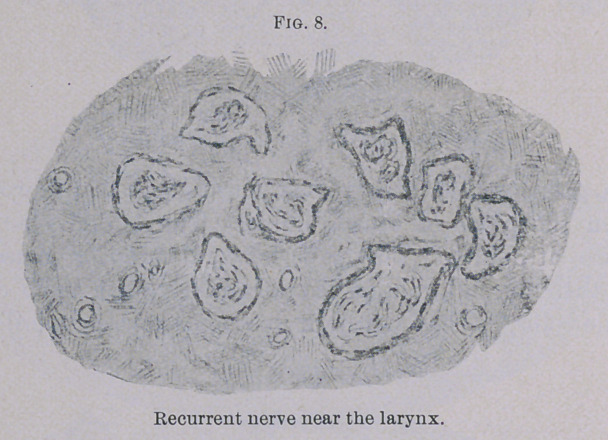


**Fig. 9. f9:**
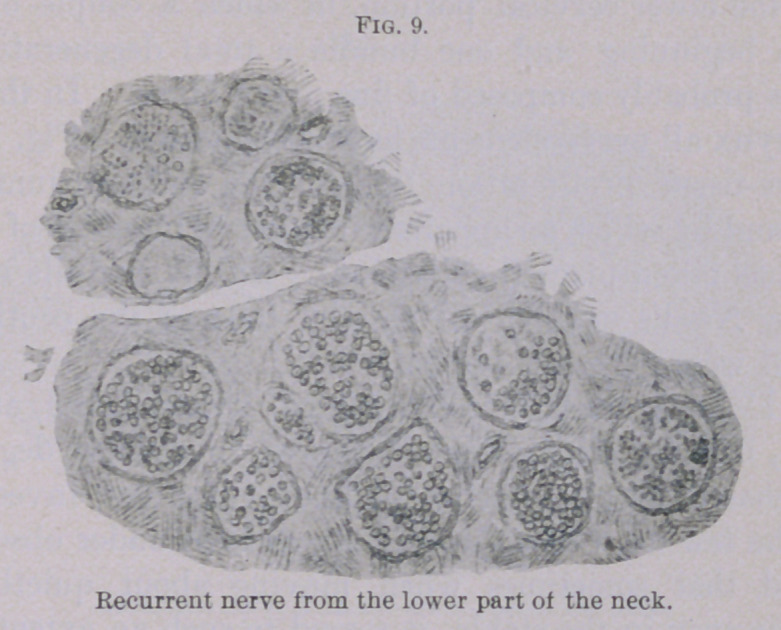


**Fig. 10. f10:**
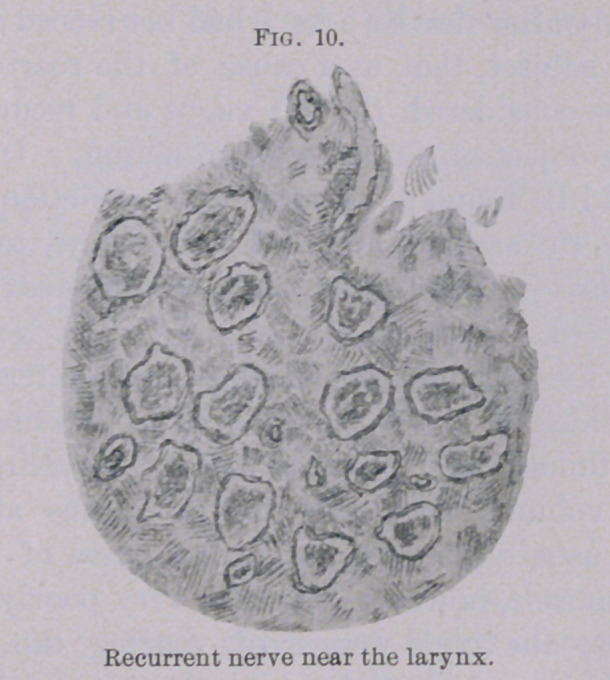


**Fig. 11. f11:**
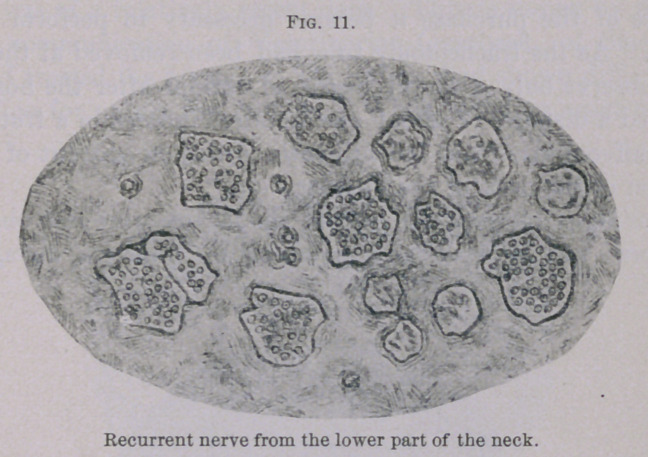


**Fig. 12. f12:**
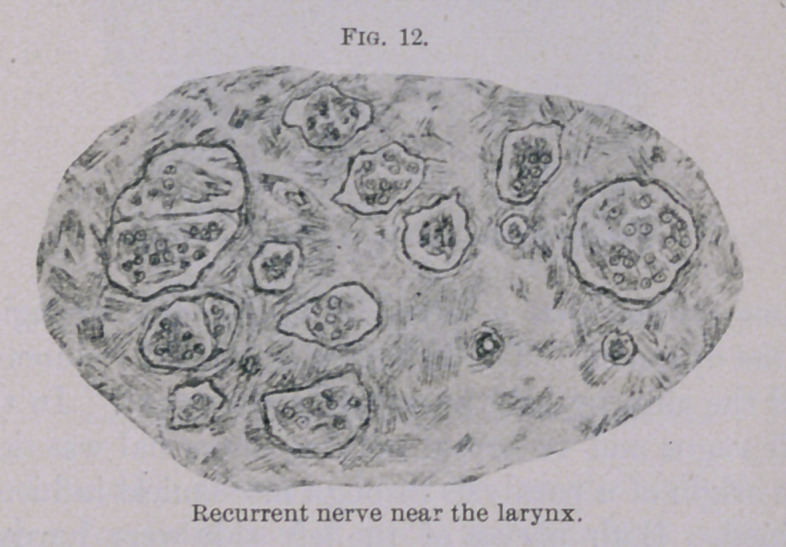


**Fig. 13. f13:**
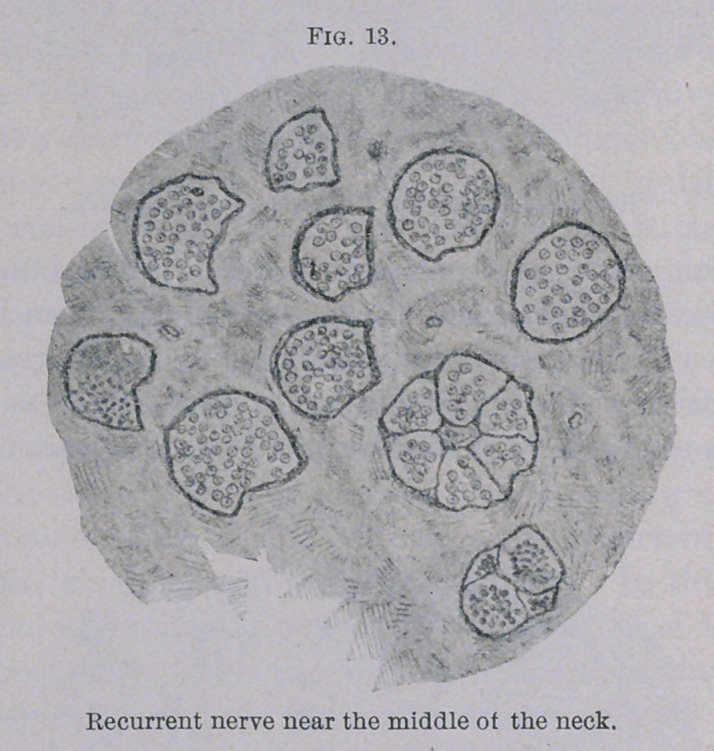


**Fig. 14. f14:**
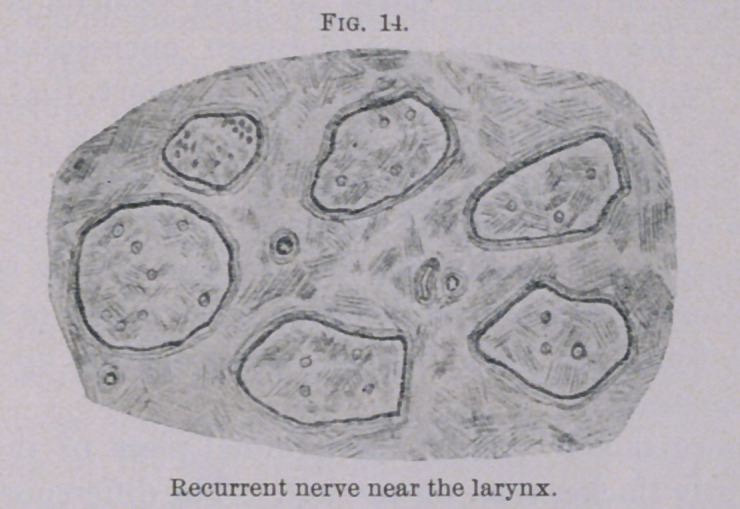


**Fig. 15. f15:**